# Supplementary material for: Proteomic Analysis in Valvular Cardiomyopathy: Aortic Regurgitation vs. Aortic Stenosis
Source: Cells. 2023 Mar 11;12(6):878. doi: 10.3390/cells12060878 (PMC10047037; doi:10.3390/cells12060878)
Supplement: Supplementary file 1 [file cells-12-00878-s001.zip › cells-2225933-supplementary.pdf]

cells

MDPI

Article

Proteomic Analysis in Valvular Cardiomyopathy: Aortic Regurgitation vs. Aortic Stenosis

Theresa Holst [1,2,†,*], Johannes Petersen [2,3,†], Sabine Ameling [4,5], Lisa Müller [1], Torsten Christ [3,6], Naomi Gedeon [2,3,6], Thomas Eschenhagen [3,6], Hermann Reichenspurner [2,3], Elke Hammer [4,5,†] and Evaldas Girdauskas [1,2,3,*,†]

1 Department of Cardiothoracic Surgery, Augsburg University Hospital, Stenglinstraße 2, 86156 Augsburg, Germany
2 Department of Cardiovascular Surgery, University Heart and Vascular Center Hamburg, Martinistraße 52, 20246 Hamburg, Germany
3 German Center of Cardiovascular Research (DZHK), Partner Site Hamburg/Kiel/Lübeck, Germany
4 Interfaculty Institute for Genetics and Functional Genomics, University Medicine Greifswald, Felix-Hausdorff-Straße 8, 17487 Greifswald, Germany
5 German Center of Cardiovascular Research (DZHK), Partner Site Greifswald, Germany
6 Institute of Experimental Pharmacology and Toxicology, University Medical Center Hamburg-Eppendorf, Martinistraße 52, 20246 Hamburg, Germany
* Correspondence: theresa.holst@uk-augsburg.de (T.H.); evaldas.girdauskas@uk-augsburg.de (E.G.); Tel.: +49-821-400-2591; Fax: +49-821-172591
† These authors contributed equally to this work.

1. Supplementary Materials

1.1. Supplementary Figure

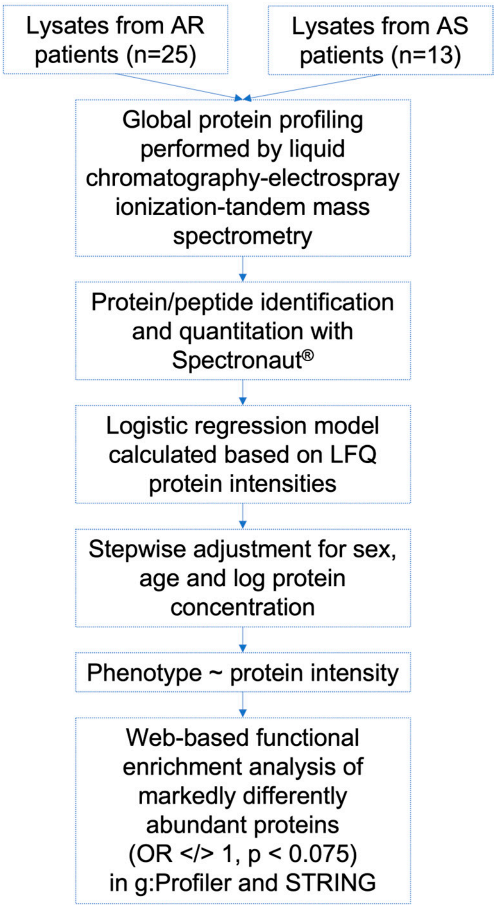

Figure S1. Flow chart for proteomic assessment of final sample set. AR: aortic regurgitation; AS: aortic stenosis; LFQ: label-free quantitation; OR: Odds ratio.

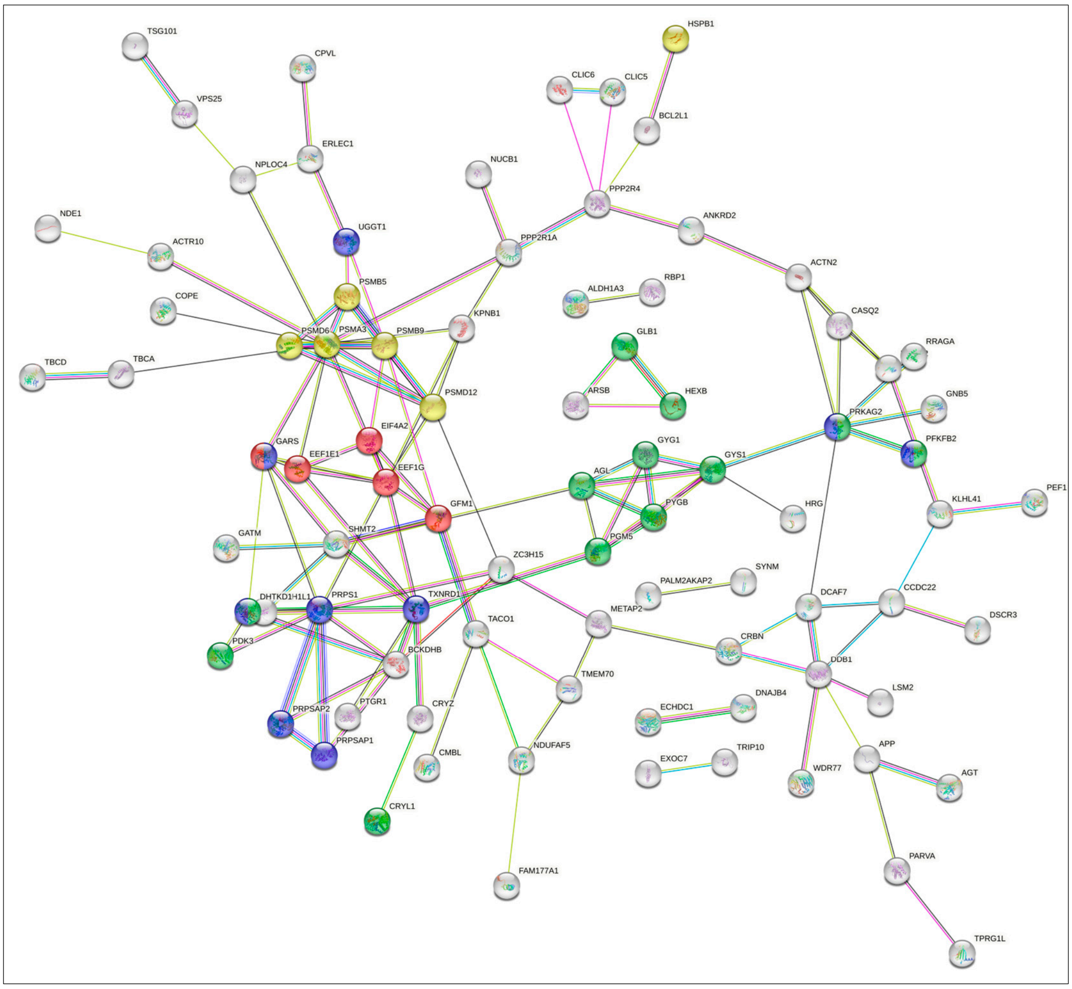

Figure S2. Network visualization of protein-protein interconnections of proteins being characteristic of the aortic regurgitation group using STRING: proteins assigned to carbohydrate metabolism (green), nucleotide metabolism (blue), protein biosynthesis (red), and the proteasome (yellow). Disconnected nodes in the network hidden.

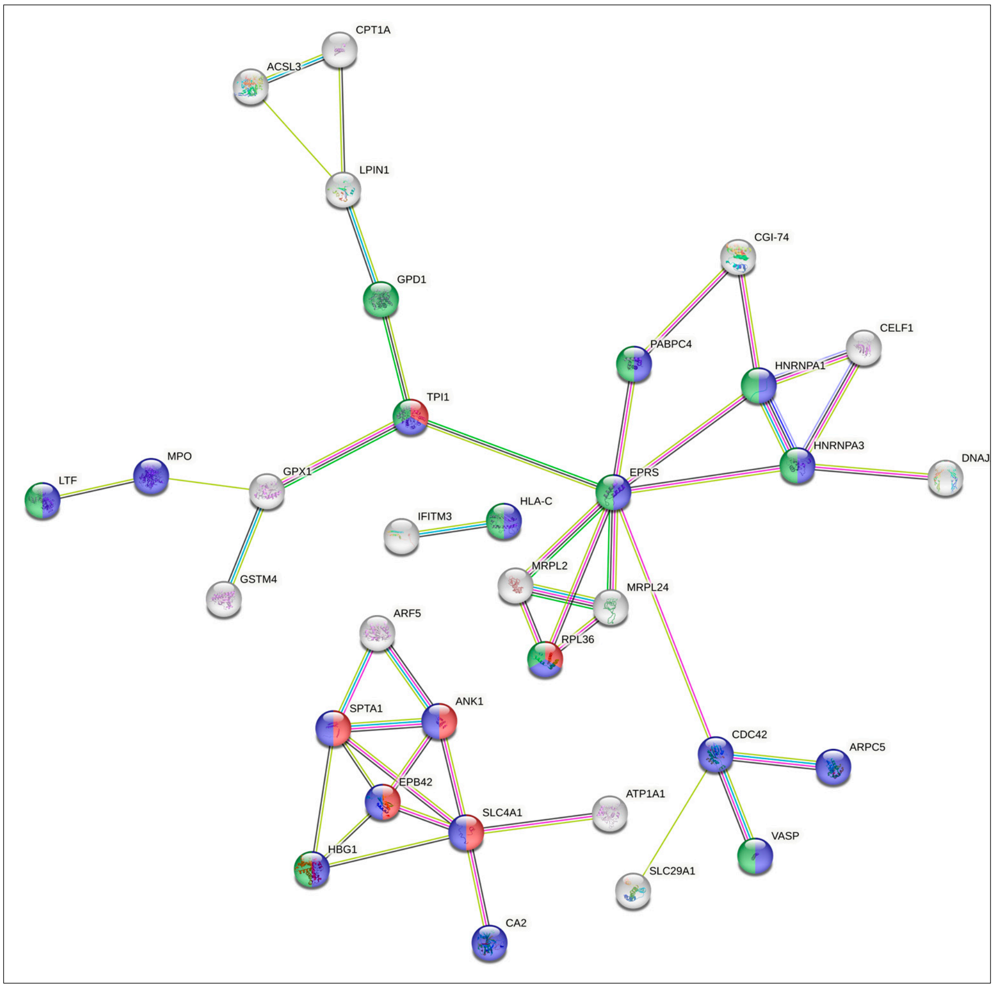

Figure S3. Network visualization of protein-protein interconnections of proteins being characteristic of the aortic stenosis using STRING: proteins assigned to the immune system (green) and hematopoietic system/angiogenesis (blue) and anemia (red) in specific. Disconnected nodes in the network hidden.

1.2. Supplementary Tables

Table S1_A. Liquid chromatography-electrospray ionization-tandem mass spectrometry parameter (data independent mode; quantitative data).

**Data independent analyses (DIA)**	
**reversed phase liquid chromatography**	**Ultimate 3000 RSLC (Thermo Scientific)**
Trap column	75 µm inner diameter, packed with 3 µm C18 particles (Acclaim PepMap100, Thermo Scientific)
Analytical column	75 µm inner diameter, packed with 2.6 µm C18 particles (Accucore, 25 cm, Thermo Scientific)
Flow rate	300 nl/min
Column oven temperature	40°C
Buffer system	Binary buffer system consisting of 0.1% acetic acid in HPLC-grade water (buffer A) and 100% ACN in 0.1% acetic acid (buffer B)
Gradient	Gradient of buffer B: 2min 2% to 5 %, 8min 5% to 7%, 60min 7% to 25%, 5min 25 to 40%, 2 min 40% to 90%, 6 min 90%, 2 min 90% to 2%, 10 min 2%
**Mass spectrometer**	**Exploris 480**
Operation mode	Data-independent
Electrospray	Nanospray Flex Ion Source
**Full MS**	
MS scan resolution	120,000
Normalized AGC target	300 %
Maximum ion injection time for the MS scan	60 ms
Scan range	350 to 1200 *m/z*
Spectra data type	Profile
**DIA scan**	
Resolution	30,000
Normalized AGC target	3000
Maximum ion injection time for the MS/MS scans	Auto
Spectra data type	Profile
Selection for MS/MS	1
Isolation window	65 windows *m/z* 13 (overlap 2)
Fixed first mass	200
Dissociation mode	Higher energy collisional dissociation (HCD)
Normalized collision energy	Stepped, 30
Dissociation mode	HCD

Table S1_B. Spectronaut parameters for peptide/protein identification and intensity extraction.

**Spectronaut 16.0.220606.53000**	

Computer Name: AGVOE-SPECTRONAUT	
User Domain Name: AGVOE-SPECTRONAUT	
User Name: spectronaut	
Analysis Mode: UI	
Analysis Type: directDIA	
Analysis Date: 05-August-2022	
Settings Used:	
**Pulsar Search\Peptides**	
Toggle N-terminal M:	True
Min Peptide Length:	7
Max Peptide Length:	52
Missed Cleavages:	2
Digest Type:	Specific
Enzymes / Cleavage Rules:	Trypsin/P
**Pulsar Search\Labeling**	
Channels:	
Channel 1:	False
Channel 2:	False
Channel 3:	False
**DIA Analysis\Data Extraction**	
MS1 Mass Tolerance Strategy:	Dynamic
Correction Factor:	1
MS2 Mass Tolerance Strategy:	Dynamic
Correction Factor:	1
Intensity Extraction MS1:	Maximum Intensity
Intensity Extraction MS2:	Maximum Intensity
**DIA Analysis\XIC Extraction**	
XIC IM Extraction Window:	Dynamic
Correction Factor:	1
XIC RT Extraction Window:	Dynamic
Correction Factor:	1
**Pulsar Search\Modifications**	
Max Variable Modifications:	5
**Database**	
Original File:	uniprot_Human_2022_01.fasta
**Select Modifications:**	
Fixed Modifications::	Carbamidomethyl (C)
Variable Modifications: :	Acetyl (Protein N-term), Oxidation (M)
**DIA Analysis\Calibration**	
MS1 Mass Tolerance Strategy:	System Default

MS2 Mass Tolerance Strategy:	System Default
Precision iRT:	True
iRT <-> RT Regression Type:	Local (Non-Linear) Regression
Exclude Deamidated Peptides:	True
MZ Extraction Strategy:	Maximum Intensity
Allow source specific iRT Calibration:	True
**<u>DIA Analysis\Identification</u>**	
Generate Decoys:	True
Decoy Limit Strategy:	Dynamic
Library Size Fraction:	0.1
Decoy Method:	Mutated
Preferred Fragment Source:	NN Predicted Fragments
Machine Learning:	Per Run
Exclude Duplicate Assays:	True
Precursor PEP Cutoff:	0.2
Protein Qvalue Cutoff (Experiment):	0.01
Protein Qvalue Cutoff (Run):	0.05
Exclude Single Hit Proteins:	False
Pvalue Estimator:	Kernel Density Estimator
Precursor Qvalue Cutoff:	0.001
Single Hit Definition:	By Stripped Sequence
**<u>DIA Analysis\Quantification</u>**	
Interference Correction:	True
MS1 Min:	2
MS2 Min:	3
Exclude All Multi-Channel Interferences:	True
Only Identified Peptides:	True
Protein LFQ Method:	Automatic
Major (Protein) Grouping:	by Protein Group Id
Minor (Peptide) Grouping:	by Stripped Sequence
Minor Group Top N:	False
Minor Group Quantity:	Sum precursor quantity
Major Group Top N:	True
Min:	2
Max:	3
Major Group Quantity:	Mean peptide quantity
Quantity MS-Level:	MS2
Quantity Type:	Area
Proteotypicity Filter:	None (set to proteotypic by in-house analysis R pipeline)
Data Filtering:	Identified in % of Runs (Percentile)
Fraction:	0.2
Imputing Strategy:	No Imputing
Cross Run Normalization:	True (local)

**DIA Analysis\PTM Workflow**	
PTM Localization:	True
Probability Cutoff:	0.75
PTM Analysis:	True
Multiplicity:	True
Run Clustering:	False
PTM Consolidation:	Sum
Flanking Region:	7
**DIA Analysis\Workflow**	
MS2 DeMultiplexing:	Automatic
Run Limit for directDIA Library:	-1
Method Evaluation:	False
Profiling Strategy:	iRT Profiling
Profiling Row Selection:	Minimum Qvalue Row Selection
Qvalue Threshold:	0.001
Profiling Target Selection:	Profile only non-identfied Precursor
Identification Criterion:	Qvalue
Threshold:	0.001
Carry-over exact Peak Boundaries:	False
Unify Peptide Peaks Strategy:	Select corresponding Peak
**DIA Analysis\Protein Inference**	
Protein Inference Workflow:	Automatic
Inference Algorithm:	IDPicker
**DIA Analysis\Post Analysis**	
Calculate Sample Correlation Matrix:	True
Calculate Explained TIC:	Quick
Differential Abundance Grouping:	Major Group (Quantification Settings)
Smallest Quantitative Unit:	Precursor Ion (Quantification Settings)
Use All MS-Level Quantities:	False
Differential Abundance Testing:	t-test
Group-Wise Testing Correction:	False
Run Clustering:	True
Distance Metric:	Manhattan Distance
Linkage Strategy:	Ward's Method
Z-score transformation:	False
Order Runs by Clustering:	True
**DIA Analysis\Pipeline Mode**	
Post Analysis Reports:	
Scoring Histograms:	True

Data Completeness Bar Chart:	True
Run Identifications Bar Chart:	True
CV Density Line Chart:	True
CVs Below X Bar Chart:	True
Generate SNE File:	True
Store Iontraces in SNE:	False
Report Schema:	C_FunGene_complex (Normal)
Reporting Unit:	Across Experiment
**Pulsar Search\Identification**	
Peptide FDR:	0.01
Protein Group FDR:	0.01
PSM FDR:	0.01
**Pulsar Search\Tolerances**	
Tolerance Parameters:	
Thermo Orbitrap:	
Calibration Search:	Dynamic
MS1 Correction Factor:	1
MS2 Correction Factor:	1
Main Search:	Dynamic
MS1 Correction Factor:	1
MS2 Correction Factor:	1
TOF:	
Calibration Search:	Dynamic
MS1 Correction Factor:	1
MS2 Correction Factor:	1
Main Search:	Dynamic
MS1 Correction Factor:	1
MS2 Correction Factor:	1
Thermo IonTrap:	
Calibration Search:	Dynamic
MS1 Correction Factor:	1
MS2 Correction Factor:	1
Main Search:	Dynamic
MS1 Correction Factor:	1
MS2 Correction Factor:	1
**Pulsar Search\Workflow**	
Use DNN Predicted Ion Mobility:	Auto
Fragment Ion Selection Strategy:	Intensity Based
In-Silico Generate Missing Channels:	False
**Pulsar Search\Result Filters**	
Precursors:	
Best N Fragments per Peptide:	True
Min:	6
Max:	10

Channel Count:	False
Modifications:	None
Amino Acids:	False
Best N Peptides per Protein Group:	False
FASTA Matched:	False
Missed Cleavage:	False
Peptide Charge:	False
Proteotypicity:	False
Fragment Ions:	
m/z :	True
Min:	300
Max:	1800
Ion Charge:	False
Ion Loss Type:	False
Ion Type:	False
Ion AA Length:	True
N:	3
Relative Intensity:	True
Min:	5

Table S2. Aortic regurgitation-associated proteins: OR > 1, $p < 0.075$. OR: Odds ratio, i.e. chance to be assigned to the aortic regurgitation group when protein intensity is high.

Protein ID	Gene name	Protein name*	OR	p-value	95% CI lower limit	95% CI upper limit
Q9UBV8	PEF1	Peflin	160.23	0.028	1.75	14708.73
Q9NVD7	PARVA	Alpha-parvin	63.56	0.052	0.97	4180.91
P46976	GYG1	Glycogenin-1	27.43	0.020	1.67	449.35
O60826	CCDC22	Coiled-coil domain-containing protein 22	26.31	0.032	1.32	525.42
O43324	EEF1E1	Eukaryotic translation elongation factor 1 epsilon-1	25.35	0.019	1.71	375.80
P30153	PPP2R1A	Serine/threonine-protein phosphatase 2A 65 kDa regulatory subunit A alpha isoform	19.04	0.051	0.99	365.84
P25686	DNAJB2	DnaJ homolog subfamily B member 2	17.56	0.043	1.10	281.40
Q14232	EIF2B1	Translation initiation factor eIF-2B subunit alpha	16.18	0.048	1.03	255.29
Q14558	PRPSAP1	Phosphoribosyl pyrophosphate synthase-associated protein 1,	15.50	0.017	1.64	146.19
O15061	SYNM	Synemin	14.64	0.021	1.51	142.18
Q15124	PGM5	Phosphoglucomutase-like protein 5	13.00	0.021	1.46	115.44
Q9NZ32	ACTR10	Actin-related protein 10	12.82	0.012	1.77	93.02
Q96IZ0	PAWR	PRKC apoptosis WT1 regulator protein	9.07	0.025	1.32	62.46
O60825	PFKFB2	6-phosphofructo-2-kinase/fructose-2,6-bisphosphatase 2	8.62	0.018	1.45	51.42
P35573	AGL	Glycogen debranching enzyme	8.45	0.026	1.28	55.62
P04196	HRG	Histidine-rich glycoprotein	8.26	0.031	1.22	56.02
Q5T0D9	TPRG1L	Tumor protein p63-regulated gene 1-like protein	8.20	0.050	1.00	67.16
Q16881	TXNRD1	Thioredoxin reductase 1, cytoplasmic	7.54	0.054	0.96	59.04
O75891	ALDH1L1	Cytosolic 10-formyltetrahydrofolate dehydrogenase	7.53	0.024	1.30	43.66
Q99766	ATP5S/DMAC2L	ATP synthase subunit s, mitochondrial	7.47	0.049	1.01	55.14

P26641	EEF1G	Elongation factor 1-gamma	7.44	0.027	1.26	44.00
Q7Z4V5	HDGFL2	Hepatoma-derived growth factor-related protein 2	7.33	0.015	1.47	36.56
P60891	PRPS1	Ribose-phosphate pyrophosphokinase 1	7.08	0.021	1.35	37.11
Q14956	GPNMB	Transmembrane glycoprotein NMB	6.86	0.011	1.56	30.25
P04792	HSPB1	Heat shock protein beta-1, HspB1	6.65	0.055	0.96	45.91
O60256	PRPSAP2	Phosphoribosyl pyrophosphate synthase-associated protein 2	6.63	0.017	1.40	31.39
P09455	RBP1	Retinol-binding protein 1	6.58	0.030	1.20	36.21
P55809	OXCT1	Succinyl-CoA:3-ketoacid coenzyme A transferase 1, mitochondrial	6.45	0.053	0.97	42.75
Q07817	BCL2L1	Bcl-2-like protein 1	6.43	0.024	1.27	32.48
Q02818	NUCB1	Nucleobindin-1	6.27	0.074	0.84	47.05
P11216	PYGB	Glycogen phosphorylase, brain form	6.26	0.038	1.11	35.33
Q96DZ1	ERLEC1	Endoplasmic reticulum lectin 1	6.15	0.074	0.84	45.01
P01019	AGT	Angiotensinogen	5.90	0.028	1.21	28.72
Q15120	PDK3	[Pyruvate dehydrogenase (acetyl-transferring)] kinase isozyme 3	5.73	0.041	1.07	30.66
P28074	PSMB5	Proteasome subunit beta type-5	5.47	0.044	1.05	28.54
Q16531	DDB1	DNA damage-binding protein 1	5.22	0.054	0.97	28.02
P35609	ACTN2	Alpha-actinin-2	5.22	0.048	1.01	26.89
Q9NYL2	MAP3K20	Mitogen-activated protein kinase kinase kinase 20	5.21	0.060	0.94	29.03
Q9Y316	MEMO1	Protein MEMO1	5.11	0.019	1.30	20.05
Q9NVT9	ARMC1	Armadillo repeat-containing protein 1	5.02	0.025	1.22	20.59
Q6VY07	PACS1	Phosphofurin acidic cluster sorting protein 1	4.97	0.029	1.18	20.91
Q9NYU2	UGGT1	UDP-glucose:glycoprotein glucosyltransferase 1	4.88	0.069	0.89	26.93
P61962	DCAF7	DDB1- and CUL4-associated factor 7	4.86	0.044	1.04	22.74
P28065	PSMB9	Proteasome subunit beta type-9	4.67	0.072	0.87	25.03
Q96HY7	DHTKD1	2-oxoadipate dehydrogenase complex component E1	4.58	0.045	1.04	20.21

Q9NXR1	NDE1	Nuclear distribution protein nudE homolog 1	4.51	0.051	0.99	20.51
Q8N8S7	ENAH	Protein enabled homolog	4.51	0.023	1.24	16.45
P05067	APP	Amyloid-beta precursor protein	4.48	0.059	0.94	21.35
P15848	ARSB	Arylsulfatase B	4.47	0.030	1.15	17.37
Q9BRG1	VPS25	Vacuolar protein-sorting-associated protein 25	4.46	0.046	1.02	19.45
Q08257	CRYZ	Quinone oxidoreductase	4.45	0.023	1.23	16.04
Q16658	FSCN1	Fascin	4.45	0.038	1.09	18.21
Q9H3G5	CPVL	Probable serine carboxypeptidase CPVL	4.44	0.033	1.12	17.54
Q9HAB8	PPCS	Phosphopantothenate--cysteine ligase, Phosphopantothenoylcysteine synthetase	4.44	0.039	1.07	18.35
P16278	GLB1	Beta-galactosidase	4.43	0.044	1.04	18.85
Q9BYV6	TRIM55	Tripartite motif-containing protein 55	4.40	0.053	0.98	19.76
O14958	CASQ2	Calsequestrin-2	4.37	0.062	0.93	20.66
Q9UPT5	EXOC7	Exocyst complex component 7	4.31	0.063	0.92	20.09
Q99816	TSG101	Tumor susceptibility gene 101 protein	4.30	0.064	0.92	20.13
O60662	KLHL41	Kelch-like protein 41	4.28	0.040	1.07	17.21
Q7L523	RRAGA	Ras-related GTP-binding protein A	4.28	0.030	1.15	15.89
P78385	KRT83	Keratin, type II cuticular Hb3	4.28	0.051	0.99	18.39
Q14974	KPNB1	Importin subunit beta-1	4.21	0.067	0.90	19.64
P08185	SERPINA6	Corticosteroid-binding globulin	4.17	0.061	0.94	18.50
Q15642	TRIP10	Cdc42-interacting protein 4	4.14	0.072	0.88	19.49
Q5TEU4	NDUFAF5	Arginine-hydroxylase NDUFAF5, mitochondrial	4.12	0.054	0.97	17.46
Q9Y2D5	AKAP2	A-kinase anchor protein 2	4.08	0.065	0.92	18.18
Q9NRK6	ABCB10	ATP-binding cassette sub-family B member 10, mitochondrial	4.06	0.061	0.94	17.61
P50579	METAP2	Methionine aminopeptidase 2	4.00	0.074	0.87	18.30
P07686	HEXB	Beta-hexosaminidase subunit beta	3.94	0.052	0.99	15.72
O00232	PSMD12	26S proteasome non-ATPase regulatory subunit 12	3.89	0.040	1.06	14.28
P05452	CLEC3B	Tetranectin	3.86	0.042	1.05	14.15
Q9NZA1	CLIC5	Chloride intracellular channel protein 5	3.85	0.068	0.90	16.43

O75347	TBCA	Tubulin-specific chaperone A	3.84	0.041	1.06	13.95
Q9Y2S2	CRYL1	Lambda-crystallin homolog	3.78	0.046	1.03	13.94
O14972	VPS26C	Vacuolar protein sorting-associated protein 26C	3.78	0.068	0.91	15.76
P25788	PSMA3	Proteasome subunit alpha type-3 (Macropain subunit C8)	3.68	0.058	0.96	14.17
Q9BSH4	TACO1	Translational activator of cytochrome c oxidase 1	3.66	0.071	0.90	14.95
Q9UGJ0	PRKAG2	5'-AMP-activated protein kinase subunit gamma-2	3.60	0.057	0.96	13.46
Q9NTX5	ECHDC1	Ethylmalonyl-CoA decarboxylase	3.60	0.053	0.99	13.12
Q9Y333	LSM2	U6 snRNA-associated Sm-like protein LSm2	3.58	0.053	0.99	13.03
P35659	DEK	Protein DEK	3.50	0.069	0.91	13.50
Q96NY7	CLIC6	Chloride intracellular channel protein 6	3.49	0.049	1.01	12.11
Q9BTW9	TBCD	Tubulin-specific chaperone D	3.48	0.029	1.14	10.65
Q9BUB7	TMEM70	Transmembrane protein 70	3.47	0.067	0.91	13.19
Q92599	SEPTIN8	Septin-8	3.47	0.036	1.08	11.10
Q14914	PTGR1	Prostaglandin reductase 1	3.45	0.036	1.09	10.97
Q3ZCW2	LGALSL	Galectin-related protein	3.42	0.040	1.06	11.04
Q96DG6	CMBL	Carboxymethylenebutenolidase homolog	3.41	0.039	1.06	10.96
Q8WU90	ZC3H15	Zinc finger CCCH domain-containing protein 15	3.41	0.036	1.08	10.74
Q9BQA1	WDR77	Methylosome protein 50	3.31	0.048	1.01	10.85
P41250	GARS1	Glycine--tRNA ligase	3.27	0.052	0.99	10.79
Q14240	EIF4A2	Eukaryotic initiation factor 4A-II	3.22	0.051	1.00	10.44
Q96RP9	GFM1	Elongation factor G, mitochondrial	3.11	0.066	0.93	10.42
Q9GZV1	ANKRD2	Ankyrin repeat domain-containing protein 2	3.09	0.054	0.98	9.72
Q15008	PSMD6	26S proteasome non-ATPase regulatory subunit 6	3.08	0.072	0.91	10.45
Q8WZA9	IRGQ	Immunity-related GTPase family Q protein	3.07	0.073	0.90	10.43
Q86TI2	DPP9	Dipeptidyl peptidase 9	3.04	0.054	0.98	9.45

Q9NPH2	ISYNA1	Inositol-3-phosphate synthase 1	3.01	0.052	0.99	9.17
P50440	GATM	Glycine amidinotransferase, mitochondrial	2.96	0.071	0.91	9.62
O14775	GNB5	Guanine nucleotide-binding protein subunit beta-5	2.96	0.069	0.92	9.53
P47895	ALDH1A3	Aldehyde dehydrogenase family 1 member A3	2.95	0.056	0.97	8.94
Q8N128	FAM177A1	Protein FAM177A1	2.91	0.068	0.92	9.18
Q9UHI8	ADAMTS1	A disintegrin and metalloproteinase with thrombospondin motifs 1	2.90	0.057	0.97	8.69
Q63HQ2	EGFLAM	Pikachurin	2.89	0.063	0.95	8.83
P21953	BCKDHB	2-oxoisovalerate dehydrogenase subunit beta, mitochondrial	2.88	0.054	0.98	8.47
P46926	GNPDA1	Glucosamine-6-phosphate isomerase 1	2.88	0.067	0.93	8.97
Q9UDY4	DNAJB4	DnaJ homolog subfamily B member 4	2.86	0.050	1.00	8.18
Q15257	PTPA	Serine/threonine-protein phosphatase 2A activator	2.86	0.067	0.93	8.79
P14324	FDPS	Farnesyl pyrophosphate synthase	2.85	0.048	1.01	8.07
P34897	SHMT2	Serine hydroxymethyltransferase, mitochondrial	2.81	0.075	0.90	8.75
Q8TAT6	NPLOC4	Nuclear protein localization protein 4 homolog	2.78	0.064	0.94	8.20
Q96SW2	CRBN	Protein cereblon	2.74	0.048	1.01	7.45
Q9P2T1	GMPR2	GMP reductase 2	2.70	0.075	0.91	8.08
Q6P5Q4	LMOD2	Leiomodin-2	2.65	0.049	1.00	7.02
P13807	GYS1	Glycogen [starch] synthase, muscle	2.49	0.059	0.97	6.43
O14579	COPE	Coatomer subunit epsilon	2.48	0.065	0.94	6.53

* Color-coding corresponding to color-coding used in Supplementary Figure S2: proteins assigned to carbohydrate metabolism (green), nucleotide metabolism (blue), protein biosynthesis (red), and the proteasome (yellow). *p*-values and Odds ratios were calculated by the *glm* function, confident intervals (95%) by the *confint.default* function of the *stats* package in R using LFQ protein intensities.

Table S3. Aortic stenosis-associated proteins: OR < 1, $p < 0.075$. OR: Odds ratio, i.e. chance to be assigned to the aortic stenosis group when protein intensity is high.

Protein ID	Gene name	Protein name*	OR	p-value	95% CI lower limit	95% CI upper limit
Q8NFW8	CMAS	N-acylneuraminate cytidylyltransferase	0.01	0.024	0.00	0.50
Q687X5	STEAP4	Metalloreductase STEAP4	0.01	0.032	0.00	0.66
Q709C8	VPS13C	Intermembrane lipid transfer protein VPS13C	0.01	0.031	0.00	0.67
Q32MK0	MYLK3	Myosin light chain kinase 3	0.06	0.028	0.00	0.74
O95573	ACSL3	Fatty acid CoA ligase Acsl3	0.06	0.047	0.00	0.96
O15511	ARPC5	Actin-related protein 2/3 complex subunit 5	0.07	0.019	0.01	0.65
Q9Y383	LUC7L2	Putative RNA-binding protein Luc7-like 2	0.10	0.042	0.01	0.92
P02549	SPTA1	Spectrin alpha chain, erythrocytic 1	0.10	0.021	0.01	0.71
P60174	TPI1	Triosephosphate isomerase	0.10	0.043	0.01	0.93
Q03013	GSTM4	Glutathione S-transferase Mu 4	0.11	0.054	0.01	1.04
P10321	HLA-C	HLA class I histocompatibility antigen, C alpha chain	0.11	0.033	0.01	0.84
Q4KMQ2	ANO6	Anoctamin-6	0.11	0.031	0.02	0.82
P00918	CA2	Carbonic anhydrase 2	0.11	0.024	0.02	0.75
Q92879	CELF1	CUGBP Elav-like family member 1	0.13	0.071	0.01	1.19
Q5T653	MRPL2	39S ribosomal protein L2, mitochondrial, L2mt, MRP-L2	0.15	0.059	0.02	1.07
P07814	EPRS1	Bifunctional glutamate/proline--tRNA ligase	0.16	0.068	0.02	1.14
P46937	YAP1	Transcriptional coactivator YAP1, Yes-associated protein 1	0.16	0.034	0.03	0.87
Q14693	LPIN1	Phosphatidate phosphatase LPIN1	0.18	0.048	0.03	0.99
Q8NE62	CHDH	Choline dehydrogenase, mitochondrial	0.19	0.056	0.04	1.04
P51991	HNRNPA3	Heterogeneous nuclear ribonucleoprotein A3	0.20	0.042	0.04	0.94
P09651	HNRNPA1	Heterogeneous nuclear ribonucleoprotein A1, hnRNP A1	0.20	0.053	0.04	1.02
Q9UBV2	SEL1L	Protein sel-1 homolog 1	0.21	0.042	0.05	0.95
P07203	GPX1	Glutathione peroxidase 1	0.22	0.044	0.05	0.96

Q96A35	MRPL24	39S ribosomal protein L24, mitochondrial	0.22	0.035	0.05	0.90
P16452	EPB42	Protein 4.2	0.22	0.032	0.06	0.88
Q9Y6C2	EMILIN1	EMILIN-1	0.22	0.035	0.06	0.90
P00414	MT-CO3	Cytochrome c oxidase subunit 3	0.23	0.042	0.05	0.95
Q8N163	CCAR2	Cell cycle and apoptosis regulator protein 2	0.23	0.065	0.05	1.09
P60953	CDC42	Cell division control protein 42 homolog	0.24	0.042	0.06	0.95
P05164	MPO	Myeloperoxidase	0.24	0.027	0.07	0.85
Q01628	IFITM3	Interferon-induced transmembrane protein 3	0.24	0.054	0.06	1.02
Q99808	SLC29A1	Equilibrative nucleoside transporter 1	0.24	0.061	0.06	1.06
P54578	USP14	Ubiquitin carboxyl-terminal hydrolase 14	0.25	0.039	0.06	0.93
Q9BQJ4	TMEM47	Transmembrane protein 47	0.25	0.028	0.07	0.86
Q13310	PABPC4	Polyadenylate-binding protein 4	0.25	0.031	0.07	0.88
P05023	ATP1A1	Sodium/potassium-transporting ATPase subunit alpha-1	0.26	0.047	0.07	0.98
P02788	LTF	Lactotransferrin	0.27	0.023	0.09	0.83
P21695	GPD1	Glycerol-3-phosphate dehydrogenase	0.28	0.070	0.07	1.11
Q9ULP0	NDRG4	Protein NDRG4	0.28	0.075	0.07	1.13
Q9Y3U8	RPL36	60S ribosomal protein L36	0.29	0.049	0.08	1.00
O60884	DNAJA2	DnaJ homolog subfamily A member 2	0.29	0.053	0.08	1.02
P42229_P51692	STAT5A	Signal transducer and activator of transcription 5A	0.30	0.047	0.09	0.98
Q14766	LTBP1	Latent-transforming growth factor beta-binding protein 1	0.30	0.049	0.09	1.00
P50552	VASP	Vasodilator-stimulated phosphoprotein	0.30	0.050	0.09	1.00
Q92629	SGCD	Delta-sarcoglycan	0.31	0.066	0.09	1.08
P50416	CPT1A	Carnitine O-palmitoyltransferase 1, liver isoform	0.31	0.041	0.10	0.95
P84085	ARF5	ADP-ribosylation factor 5	0.31	0.056	0.09	1.03
Q9NZ45	CISD1	CDGSH iron-sulfur domain-containing protein 1	0.32	0.053	0.10	1.02
Q8NBX0	SCCPDH	Saccharopine dehydrogenase-like oxidoreductase	0.33	0.059	0.11	1.04

P16157	ANK1	Ankyrin-1	0.34	0.046	0.12	0.98
P09382	LGALS1	Galectin-1	0.35	0.057	0.12	1.03
P20336	RAB3A	Ras-related protein Rab-3A	0.38	0.055	0.14	1.02
P02730	SLC4A1	Band 3 anion transport protein	0.39	0.075	0.14	1.10
P69891	HBG1	Hemoglobin subunit gamma-1	0.39	0.066	0.15	1.06

* Color-coding corresponding to color-coding used in Supplementary Figure S3: proteins assigned to the immune system (green) and hematopoietic system/angiogenesis (blue) and anemia (red) in specific. *p*-values and Odds ratios were calculated by the *glm* function, confident intervals (95%) by the *confint.default* function of the *stats* package in R using LFQ protein intensities.

Table S4. Results of g:Profiler enrichment analysis of proteins with $p < 0.075$ in the logistic regression analysis. Categories with adjusted *p*-value < 0.05 (calculated by the g:SCS approach in g:Profiler) in either one or both analyses of proteins with Odds ratio > 1 or Odds ratio < 1 shown.

	term_name	term_id	term_size (no of mole-cules in category)	adj pValue (AR)	-log10adj pValue (AR)	list size (AR)	proteins with OR >1 (AR)	adj pValue (AS)	-log10adj pValue (AS)	list size (AS)	proteins with OR <1 (AS)
GO:BP	organonitrogen compound metabolic process	GO:1901564	6522	9.9E-05	4.00	114	63				
GO:BP	small molecule metabolic process	GO:0044281	1837	1.7E-04	3.77	114	29				
GO:BP	catabolic process	GO:0009056	2587	2.5E-04	3.60	114	35				
GO:BP	cellular catabolic process	GO:0044248	2121	1.0E-03	2.98	114	30				
GO:BP	nucleobase-containing small molecule meta-bolic process	GO:0055086	674	1.2E-03	2.92	114	16				
GO:BP	carbohydrate metabolic process	GO:0005975	594	1.3E-03	2.89	114	15				
GO:BP	carbohydrate catabolic process	GO:0016052	154	3.3E-03	2.48	114	8				
GO:BP	organic substance catabolic process	GO:1901575	2128	3.6E-03	2.44	114	29				
GO:BP	5-phosphoribose 1-diphosphate biosynthetic process	GO:0006015	6	5.2E-03	2.28	114	3				
GO:BP	5-phosphoribose 1-diphosphate metabolic process	GO:0046391	6	5.2E-03	2.28	114	3				
GO:BP	generation of precursor metabolites and en-ergy	GO:0006091	505	6.0E-03	2.22	114	13				
GO:BP	cellular carbohydrate metabolic process	GO:0044262	291	7.1E-03	2.15	114	10				

GO:BP	negative regulation of molecular function	GO:0044092	1111	1.3E-02	1.88	114	19				
GO:BP	purine-containing compound biosynthetic process	GO:0072522	251	1.6E-02	1.81	114	9				
GO:BP	cellular macromolecule metabolic process	GO:0044260	3234	1.7E-02	1.76	114	36				
GO:BP	purine-containing compound metabolic process	GO:0072521	506	3.3E-02	1.48	114	12				
GO:BP	nucleotide metabolic process	GO:0009117	597	3.6E-02	1.44	114	13				
GO:BP	proteolysis	GO:0006508	1783	4.1E-02	1.38	114	24				
GO:BP	nucleoside phosphate metabolic process	GO:0006753	607	4.3E-02	1.37	114	13				
GO:BP	organic acid metabolic process	GO:0006082	958					8.8E-03	2.06	54	12
GO:BP	response to stress	GO:0006950	3938					2.5E-02	1.60	54	24
GO:BP	response to chemical	GO:0042221	4383					4.9E-02	1.31	54	25
GO:BP	oxoacid metabolic process	GO:0043436	951					5.0E-02	1.30	54	11
GO:BP	regulation of biological quality	GO:0065008	3726					9.5E-03	2.02	54	24
GO:BP	cellular response to chemical stimulus	GO:0070887	3049					1.9E-02	1.73	54	21
GO:CC	cytoplasm	GO:0005737	12270	6.6E-19	18.18	114	109	6.5E-05	4.19	54	48
GO:CC	cytosol	GO:0005829	5419	5.3E-12	11.28	114	67				
GO:CC	extracellular exosome	GO:0070062	2108	9.0E-12	11.05	114	41	1.6E-05	4.80	54	20
GO:CC	extracellular vesicle	GO:1903561	2132	1.3E-11	10.88	114	41	3.3E-06	5.48	54	21
GO:CC	extracellular organelle	GO:0043230	2133	1.3E-11	10.87	114	41	3.3E-06	5.48	54	21
GO:CC	extracellular membrane-bounded organelle	GO:0065010	2133	1.3E-11	10.87	114	41	3.3E-06	5.48	54	21
GO:CC	vesicle	GO:0031982	3973	1.5E-09	8.82	114	53	7.6E-03	2.12	54	23
GO:CC	secretory granule lumen	GO:0034774	320	4.4E-07	6.36	114	14				
GO:CC	cytoplasmic vesicle lumen	GO:0060205	323	5.0E-07	6.30	114	14				

GO:CC	vesicle lumen	GO:0031983	325	5.4E-07	6.27	114	14						
GO:CC	extracellular space	GO:0005615	3368	2.0E-06	5.70	114	43	2.3E-02	1.63	54	20		
GO:CC	extracellular region	GO:0005576	4302	4.4E-06	5.36	114	49	8.5E-03	2.07	54	24		
GO:CC	catalytic complex	GO:1902494	1707	1.3E-05	4.88	114	28						
GO:CC	proteasome complex	GO:0000502	66	1.7E-05	4.77	114	7						
GO:CC	ficolin-1-rich granule lumen	GO:1904813	124	9.2E-05	4.04	114	8						
GO:CC	endopeptidase complex	GO:1905369	88	1.3E-04	3.90	114	7						
GO:CC	intracellular anatomical structure	GO:0005622	17820	1.5E-04	3.82	114	111						
GO:CC	contractile fiber	GO:0043292	247	2.2E-04	3.65	114	10						
GO:CC	ribose phosphate diphosphokinase complex	GO:0002189	5	4.3E-04	3.37	114	3						
GO:CC	peptidase complex	GO:1905368	122	1.1E-03	2.94	114	7						
GO:CC	myofibril	GO:0030016	238	1.4E-03	2.84	114	9						
GO:CC	ficolin-1-rich granule	GO:0101002	184	1.8E-03	2.75	114	8						
GO:CC	secretory vesicle	GO:0099503	1043	2.5E-03	2.60	114	18						
GO:CC	membrane-enclosed lumen	GO:0031974	6637	3.4E-03	2.47	114	57						
GO:CC	organelle lumen	GO:0043233	6637	3.4E-03	2.47	114	57						
GO:CC	intracellular organelle lumen	GO:0070013	6637	3.4E-03	2.47	114	57						
GO:CC	sarcomere	GO:0030017	217	6.0E-03	2.22	114	8						
GO:CC	supramolecular fiber	GO:0099512	1017	6.9E-03	2.16	114	17						
GO:CC	supramolecular polymer	GO:0099081	1026	7.8E-03	2.11	114	17						
GO:CC	secretory granule	GO:0030141	870	1.6E-02	1.81	114	15						
GO:CC	organelle	GO:0043226	16770	2.5E-02	1.60	114	104						
GO:CC	cytoskeleton	GO:0005856	2381	2.6E-02	1.59	114	27						
GO:CC	supramolecular complex	GO:0099080	1377	3.0E-02	1.52	114	19						
GO:CC	azurophil granule lumen	GO:0035578	91	3.6E-02	1.44	114	5						
GO:CC	I band	GO:0031674	146	3.8E-02	1.43	114	6						

GO:CC	mitochondrion	GO:0005739	1684					4.7E-02	1.32	54	13
GO:CC	mitochondrial envelope	GO:0005740	807					3.8E-02	1.42	54	9
GO:CC	mitochondrial outer membrane	GO:0005741	211					4.4E-02	1.36	54	5
GO:CC	membrane	GO:0016020	9856					1.5E-02	1.83	54	39
GO:CC	mitochondrial membrane	GO:0031966	756					2.3E-02	1.64	54	9
GO:CC	phagocytic vesicle lumen	GO:0097013	5					1.5E-02	1.83	54	2
GO:MF	protein binding	GO:0005515	14832	5.3E-06	5.28	117	110				
GO:MF	identical protein binding	GO:0042802	2121	4.5E-04	3.35	117	31				
GO:MF	ribose phosphate diphosphokinase activity	GO:0004749	5	9.4E-04	3.03	117	3				
GO:MF	diphosphotransferase activity	GO:0016778	6	1.9E-03	2.73	117	3				
GO:MF	protein homodimerization activity	GO:0042803	701	6.6E-03	2.18	117	15				
GO:MF	UDP-glucosyltransferase activity	GO:0035251	12	2.0E-02	1.70	117	3				
GO:MF	protein dimerization activity	GO:0046983	1088	2.7E-02	1.57	117	18				
KEGG	Proteasome	KEGG:03050	46	2.1E-03	2.67	63	5				
KEGG	Starch and sucrose metabolism	KEGG:00500	36	1.3E-02	1.88	63	4				
KEGG	Metabolic pathways	KEGG:01100	1535	2.5E-02	1.60	63	24				
KEGG	Glycosaminoglycan degradation	KEGG:00531	19	3.2E-02	1.49	63	3				
REAC	Metabolism	REAC:R-HSA-1430728	2075	2.2E-06	5.66	86	41				
REAC	Metabolism of carbohydrates	REAC:R-HSA-71387	284	1.4E-03	2.87	86	12				
REAC	AUF1 (hnRNP D0) binds and destabilizes mRNA	REAC:R-HSA-450408	54	2.0E-03	2.69	86	6				

REAC	Hh mutants are degraded by ERAD	REAC:R-HSA-5362768	56	2.5E-03	2.60	86	6
REAC	Hh mutants abrogate ligand secretion	REAC:R-HSA-5387390	59	3.5E-03	2.46	86	6
REAC	Defective CFTR causes cystic fibrosis	REAC:R-HSA-5678895	61	4.2E-03	2.38	86	6
REAC	Hedgehog ligand biogenesis	REAC:R-HSA-5358346	65	6.1E-03	2.22	86	6
REAC	ABC-family proteins mediated transport	REAC:R-HSA-382556	103	8.7E-03	2.06	86	7
REAC	ABC transporter disorders	REAC:R-HSA-5619084	78	1.7E-02	1.76	86	6
REAC	Cross-presentation of soluble exogenous antigens (endosomes)	REAC:R-HSA-1236978	49	2.0E-02	1.70	86	5
REAC	Regulation of activated PAK-2p34 by proteasome mediated degradation	REAC:R-HSA-211733	50	2.2E-02	1.65	86	5
REAC	Regulation of ornithine decarboxylase (ODC)	REAC:R-HSA-350562	51	2.4E-02	1.61	86	5
REAC	Degradation of beta-catenin by the destruction complex	REAC:R-HSA-195253	83	2.5E-02	1.61	86	6

REAC	Vpu mediated degradation of CD4	REAC:R-HSA-180534	52	2.7E-02	1.57	86	5
REAC	Autodegradation of the E3 ubiquitin ligase COP1	REAC:R-HSA-349425	52	2.7E-02	1.57	86	5
REAC	Ubiquitin Mediated Degradation of Phosphorylated Cdc25A	REAC:R-HSA-69601	52	2.7E-02	1.57	86	5
REAC	p53-Independent DNA Damage Response	REAC:R-HSA-69610	52	2.7E-02	1.57	86	5
REAC	p53-Independent G1/S DNA damage checkpoint	REAC:R-HSA-69613	52	2.7E-02	1.57	86	5
REAC	Ubiquitin-dependent degradation of Cyclin D	REAC:R-HSA-75815	52	2.7E-02	1.57	86	5
REAC	Glycogen metabolism	REAC:R-HSA-8982491	27	2.7E-02	1.57	86	4
REAC	Glycogen storage disease type XV (GYG1)	REAC:R-HSA-3814836	2	2.8E-02	1.55	86	2
REAC	Glycogen storage disease type 0 (muscle GYS1)	REAC:R-HSA-3828062	2	2.8E-02	1.55	86	2
REAC	Regulation of Apoptosis	REAC:R-HSA-169911	53	3.0E-02	1.53	86	5
REAC	Vif-mediated degradation of APOBEC3G	REAC:R-HSA-180585	53	3.0E-02	1.53	86	5
REAC	Negative regulation of NOTCH4 signaling	REAC:R-HSA-9604323	53	3.0E-02	1.53	86	5

REAC	Regulation of mRNA stability by proteins that bind AU-rich elements	REAC:R-HSA-450531	86	3.0E-02	1.52	86	6
REAC	Host Interactions of HIV factors	REAC:R-HSA-162909	125	3.0E-02	1.52	86	7
REAC	Regulation of RUNX3 expression and activity	REAC:R-HSA-8941858	54	3.2E-02	1.49	86	5
REAC	SCF-beta-TrCP mediated degradation of Emi1	REAC:R-HSA-174113	55	3.5E-02	1.45	86	5
REAC	Degradation of AXIN	REAC:R-HSA-4641257	55	3.5E-02	1.45	86	5
REAC	FBXL7 down-regulates AURKA during mitotic entry and in early mitosis	REAC:R-HSA-8854050	55	3.5E-02	1.45	86	5
REAC	MAPK6/MAPK4 signaling	REAC:R-HSA-5687128	89	3.7E-02	1.44	86	6
REAC	Degradation of DVL	REAC:R-HSA-4641258	57	4.2E-02	1.38	86	5
REAC	Stabilization of p53	REAC:R-HSA-69541	57	4.2E-02	1.38	86	5
REAC	Metabolism of polyamines	REAC:R-HSA-351202	58	4.6E-02	1.34	86	5

REAC	NIK-->noncanonical NF-kB signaling	REAC:R-HSA-5676590	59	5.0E-02	1.30	86	5
